# Lymphopenia at diagnosis is highly prevalent in myelodysplastic syndromes and has an independent negative prognostic value in IPSS-R-low-risk patients

**DOI:** 10.1038/s41408-019-0223-7

**Published:** 2019-08-09

**Authors:** Tobias Silzle, Sabine Blum, Esther Schuler, Jennifer Kaivers, Martina Rudelius, Barbara Hildebrandt, Norbert Gattermann, Rainer Haas, Ulrich Germing

**Affiliations:** 1 Department of Medical Oncology and Hematology, Cantonal Hospital St. Gallen, St. Gallen, Switzerland; 2 Service and Central Laboratory of Hematology, University Hospital of Lausanne, Lausanne, Switzerland; 3 Department of Hematology, Oncology, and Clinical Immunology, Heinrich Heine University Düsseldorf, Düsseldorf, Germany; 4 Institute of Pathology, Ludwig-Maximilians-University Munich, Munich, Germany; 5 Department of Human Genetics, Heinrich Heine University Düsseldorf, Düsseldorf, Germany

**Keywords:** Myelodysplastic syndrome, Risk factors

## Abstract

Lymphopenia is associated with an increased mortality in several medical conditions. Its prognostic impact in myelodysplastic syndromes (MDS) is less well studied. Hence, we analyzed 1023 patients from the Düsseldorf MDS-registry with regard to the absolute lymphocyte count (ALC) at diagnosis. An ALC below the median of the population (1.2 × 10^9^/l) was associated with lower counts of neutrophils (median 1.35 vs. 1.92 × 10^9^/l, *p* < 0.001) and platelets (median 100 vs. 138 × 10^9^/l, *p* < 0.001) and with a significant lower overall survival in univariate analysis (whole cohort: median 36 vs. 46 months, *p* = 0.016; 721 patients without hematopoietic stem cell transplantation or induction chemotherapy: median 36 vs. 56 months, *p* = 0.001). For low-risk MDS according to IPSS-R, an ALC < 1.2 × 10^9^/l was of additional prognostic value in a multivariate Cox regression model together with age (< or ≥65 years) and LDH (< or ≥normal value of 240 U/l; HR 1.46, 95% CI: 1.03–2.08, *p* = 0.033). These data support the hypothesis of subtle but clinical relevant changes of the adaptive immune system in MDS. Further studies are necessary to identify the ALC cut-off best suitable for prognostication and the mechanisms responsible for the impairment of lymphoid homeostasis in MDS.

## Introduction

The myelodysplastic syndromes (MDS) comprise a heterogeneous group of hematological stem cell disorders arising primarily in the elderly population. Genetic and epigenetic changes in hematopoietic stem cells and alterations in the hematopoietic niche are the main factors that render hematopoiesis ineffective and increase the risk of leukemic transformation due to increased genetic instability.

An accurate risk stratification regarding the latter event is essential for newly diagnosed MDS patients. The most frequently used score for risk stratification, the international prognostic scoring system (IPSS)^1^ and its revised version^2^ take the degree of cytopenias, the bone marrow blast count and presence and type of cytogenetic changes determined by conventional metaphase cytogenetics into account. Higher-risk MDS patients are candidates for allogeneic hematopoietic stem cell transplantation (HSCT) because of the risk of leukemic transformation and a very short overall survival (OS) even without transformation. For lower-risk MDS patients best supportive care and improvement of cytopenias are the cornerstones of therapy. Additional risk factors such as elevated lactate-dehydrogenase (LDH), bone marrow fibrosis, and recently more and more recognized the mutational profile as determined by next generation sequencing (NGS) are important additional factors that can be considered for clinical decision making, especially in intermediate risk patients according to the IPSS-R^3^.

A low-lymphocyte count is a prognostic factor in solid tumors and lymphoid malignancies^4^. The prevalence and prognostic impact of lymphopenia in MDS patients has been studied less systematically. We, therefore, conducted this study to provide additional evidence with a focus on MDS subtypes according to the WHO 2016 classification, the IPSS-R and the presence of comorbidities.

## Material and methods

We screened the Düsseldorf MDS-registry for patients with information about the absolute lymphocyte count (ALC) at diagnosis and the IPSS-R category. The Düsseldorf MDS-registry covers virtually all newly diagnosed MDS-cases in the Greater Düsseldorf Area since 1982. Structure of the registry and the diagnostic criteria for inclusion of cases have been described earlier in detail^5^. The local ethics committee (University Hospital, Heinrich Heine University, Düsseldorf) approved the study, which followed the 2000 revision of the Declaration of Helsinki. All patients gave their written informed consent before being included into the Düsseldorf MDS registry.

Patients with an ALC > 5.0 × 10^9^/l were excluded from the dataset, since they could suffer from a concomitant lymphoproliferative disorder, as were cases with a white blood cell count > 13.0 × 10^9^/l, since they could represent myelodys plastic/myeloproliferative neoplasms. For stratification of patients into lymphopenic and nonlymphopenic, a cut-off of <1.0 × 10^9^/l lymphocytes was used, according to the normal range of our institutional laboratory (1.0–4.4 × 10^9^/l). For survival analyses, an ALC of 1.2 × 10^9^/l was used as an additional discriminator, since this value was of prognostic relevance in earlier studies^6–8^ and represented the median of our cohort. Data for the MDS comorbidity index (MDS-CI) were retrieved from the dataset of a study published previously^9^.

Categorical variables were analyzed by frequency tables and compared by the *χ*^2^-test, continuous variables were described by median (range) and compared between different groups by the Mann–Whitney (comparison of two groups) or the Kruskal–Wallis test (comparison of ≥3 groups), since data did not follow a normal distribution. Overall and leukemia-free survival were calculated in months from the date of diagnosis to the respective event date. Time-to-event-curves were calculated by the Kaplan–Meier method and the log-rank test was used for univariate comparison. Cox proportional hazard regression model was applied for multivariate analysis. All statistical tests were two-sided and a *p* value < 0.05 was considered statistically significant. All analyses were performed with IBM SPSS Statistics, Version 25.

## Results

### Study population

A total of 1023 patients (male *n* = 598 [58%]; female *n* = 425 [42%] with a median age of 66 years (range: 15–91) were identified. The median follow-up was 28 months (range: 0–500). Totally, 645 patients (63%) died during the follow-up, a leukemic transformation occurred in 254 patients (25%). In all, 117 cases (11%) were therapy-related following cytotoxic treatment or radiation therapy for another malignancy or a benign disorder, seven cases (0.6%) were secondary MDS evolving from another hematological neoplasm.

Totally, 133 patients (13%) underwent HSCT and 81 (8%) were treated with at least one course of an intensive induction chemotherapy. The remaining patients received other disease modifying treatments like immunomodulatory drugs (Lenalidomide or Thalidomide, *n* = 62) or hypomethylating agents (Azacytidine, *n* = 76, Decitabine *n* = 12), were treated with low-intensity treatments like erythropoiesis stimulating agents or immunosuppression (cyclosporine ± anti-thymocyte globulin) or received best supportive care, including transfusion and iron chelation.

Distribution of MDS subtypes according to the WHO 2016 classification, cytogenetic risk groups according to the IPSS-R and the IPSS-Risk categories, as well as peripheral blood values, blast counts in bone marrow and peripheral blood and additional parameters (LDH, presence or absence of bone marrow fibrosis, transfusion dependency) at the time point of diagnosis are listed in detail in Table 1.

**Table 1:** Characteristics of the study population and comparison of several disease characteristics after stratification according to an absolute lymphocyte count < or ≥1.2 × 10^9^/l

	Whole population (*n* = 1023)	ALC < 1.2 × 10^9^/l (*n* = 502)	ALC ≥ 1.2 × 10^9^/l (*n* = 521)	*p* Value
*WHO 2016, n (%)*				
MDS-SLD	75 (7.3)	44 (8.8)	31 (6.0)	
MDS-MLD	311 (30.4)	172 (34.3)	139 (26.7)	
MDS-RS-SLD	47 (4.6)	16 (3.2)	31 (6.0)	
MDS-RS-MLD	93 (9.1)	37 (7.4)	56 (10.7)	
MDS(del5q)	115 (11.2)	42 (8.4)	73 (14.0)	
MDS-EB-1	167 (16.3)	70 (13.9)	97 (18.6)	
MDS-EB-2	203 (19.8)	114 (22.7)	89 (17.1)	
MDS-U	12 (1.2)	7 (1.4)	5 (1.0)	
*IPSS-R, n (%)*				
Very low	58 (5.7)	24 (4.8)	34 (6.5)	
Low	325 (31.8)	148 (29.5)	177 (34)	
Intermediate	263 (25.7)	126 (25.1)	137 (26.3)	
High	188 (18.4)	92 (18.3)	96 (18.4)	
Very high	189 (18.5)	112 (22.3)	77 (14.8)	
*IPSS-R cytogenetic risk group, n (%)*				
Very good	36 (3,5)	19 (3.8)	17 (3.3)	
Good	624 (61)	287 (57.2)	337 (64.7)	
Intermediate	171 (16.7)	92 (18.3)	79 (15.2)	
Poor	74 (7.2)	43 (8.6)	31 (6)	
Very poor	118 (11.5)	61 (12.2)	57 (10.9)	
Age median [years] (range)	66 (15–91)	67 (17–91)	65 (15–86)	0.076
*Gender, n (%)*				
Male	598 (58.5)	310 (61.8)	288 (55.3)	0.036
Leukemic transformation, *n* (%)	254 (24.8)	119 (23.7)	135 (25.9)	
Deaths, *n* (%)	645 (63)	319 (63,5)	326 (62.6)	
Lost to follow-up, *n* (%)	35 (3.4)	22 (4.4)	13 (2.5)	
Hemoglobin, median [g/l] (range)	95 (3–16.9)	93 (3.7–16.9)	97 (3–16.6)	0.04
Neutrophil count, median [×10^9^/l] (range)	1.62 (0–10.59)	1.35 (0.26–10.59)	1.92 (0–10.24)	<0.001
Platelet count, median [×10^9^/l] (range)	116 (2–1540)	100 (3–987)	138 (2–1540)	<0.001
*LDH*				
Data available, *n* (%)	869 (84.9)	438 (87.3)	431 (82.7)	
LDH, median [U/l] (range)	200 (49–1848)	207 (76–900)	197 (49–1848)	0.049
LDH > ULN [240 U/l], *n* (%)	274 (31.5)	152 (34.7)	122 (28.3)	0.056
Blasts bone marrow, median [%] (range)	3 (0–19)	3 (0–19)	3 (0–19)	0.577
Blasts peripheral blood, median [%] (range)	0 (0–19)	0 (0–19)	0 (0–19)	0.235
Bone marrow fibrosis data available, *n* (%)	558 (54.5)	262 (52.2)	296 (56.8)	
With bone marrow fibrosis, *n* (%)	79 (14.2)	31 (11.8)	48 (16.2)	0.146
*Transfusion-dependency*				
Data available, *n* (%)	425 (41.5)	205 (40.8)	220 (42.2)	
Transfusion dependent, *n* (%)	223 (52.5)	119 (58)	104 (47)	0.032

### ALC in the study population, influence of gender, age, and comorbidities

The median ALC of the whole population was 1.22 × 10^9^/l (range: 0.002–4.93). There was no statistically significant difference in ALC between patients aged 65 years or older as compared to 64 years or younger (median 1.18 × 10^9^/l vs. 1.24 × 10^9^/l, *p* = 0.155) or between patients within the first (15–58 years) and fourth (74–91 years) quartile of age (median ALC 1.22 × 10^9^/l vs. 1.10 × 10^9^/l, *p* = 0.076). Male patients had significantly lower ALC than female patients, albeit with a small difference in terms of absolute numbers (median 1.17 × 10^9^/l vs. 1.27 × 10^9^/l, *p* = 0.027).

The MDS-CI was available in 350 patients, with 200 patients (57%) in the low-risk (score 0), 118 patients (34%) in the intermediate- (score 1–2) and 32 patients (9%) in the high-risk category (score > 2). No differences of the ALC were detected between the different MDS-CI categories (median ALC 1.26 × 10^9^/l, 1.19 × 10^9^/l and 1.16 × 10^9^/l, respectively, *p* = 0.258).

### Lymphocyte counts in different MDS subgroups

Despite considerable inter-individual variations of the ALC, significant differences were detected between specific MDS subgroups, with a moderate extent in terms of absolute numbers (see Table 2).

**Table 2:** Distribution of the absolute lymphocyte count in different MDS subtypes

	*n*	Median absolute lymphocyte count [×10^9^/l]	SD	Range	*p* Value
Whole population	1023	1.22	0.76	0.02–4.93	
*MDS subgroups according to WHO 2016*					<0.001
MDS-SLD	75	1.08	0.65	0.07–4.08	
MDS-MLD	311	1.11	0.78	0.09–4.93	
MDS-RS-SLD	47	1.49	0.74	0.44–3.55	
MDS-RS-MLD	93	1.38	0.80	0.02–3.98	
MDSdel(5q)	115	1.34	0.62	0.12–3.46	
MDS-EB-1	167	1.32	0.70	0.08–3.60	
MDS-EB-2	203	1.14	0.83	0.05–4.93	
MDS-U	12	1.08	0.94	0.31–3.43	
*MDS subgroups according to IPSS-R*					0.034
IPSS-R very low	58	1.34	0.72	0.22–3.98	
IPSS-R low	325	1.27	0.71	0.10–4.09	
IPSS-R intermediate	263	1.22	0.77	0.02–4.93	
IPSS-R high	188	1.24	0.76	0.08–4.93	
IPSS-R very high	189	1.05	0.84	0.05–4.37	
MDS with excess blasts	370	1.20	0.78	0.05–4.93	
MDS without excess blasts	653	1.22	0.75	0.02–4.93	0.617
MDS del(5q)	115	1.34	0.62	0.12–3.46	
MDS non-del(5q)	908	1.19	0.77	0.02–4.93	0.054
MDS-SLD/MLD	385	1.09	0.75	0.07–4.93	
MDS-RS-SLD/MLD	140	1.43	0.78	0.02–3.98	<0.001
Lower risk MDS (IPSS-R very low/low)	640	1.17	0.79	0.02–4.93	
Higher risk MDS (IPSS-R intermediate/high/very high)	383	1.28	0.71	0.10–4.09	0.009
Therapy-related MDS	117	1.06	0.76	0.02–4.93	
Primary MDS	899	1.24	0.76	0.05–4.93	0.006
Transfusion dependent	223	1.15	0.78	0.02–4.37	
Transfusion independent	202	1.26	0.71	0.09–3.63	0.015
With bone marrow fibrosis	79	1.31	0.85	0.18–4.37	
Without bone marrow fibrosis	479	1.22	0.78	0.02–4.93	0.533

With regard to the WHO2016 classification, the ALC was significantly higher in MDS with ring sideroblasts and single or multilineage dysplasia (MDS-RS-SLD, MDS-RS-MLD) as compared to MDS with single or multilineage dysplasia without ring sideroblasts (MDS-SLD, MDS-MLD; median 1.43 × 10^9^/l vs. 1.09 × 10^9^/l, *p* < 0.001), without a significant difference between MDS-RS-SLD and MDS-RS-MLD. Cases with primary MDS showed higher ALCs than therapy-related MDS (median 1.24 × 10^9^/l vs. 1.06 × 10^9^/l, *p* = 0.006). For MDS with isolated del(5q) as compared to the other subtypes, the difference was not significant (median ALC 1.34 × 10^9^/l vs. 1.19 × 10^9^/l, *p* = 0.054). MDS with excess blasts (MDS-EB-1 and MDS-EB-2) did not differ from those without excess blasts.

The ALC was lower in patients with higher-risk MDS (IPSS-R intermediate, high and very high) compared to lower-risk MDS (IPSS-R very low and low; median 1.17 × 10^9^/l vs. 1.28 × 10^9^/l, *p* = 0.009).

The transfusion status at diagnosis was available for 425 patients. Transfusion-dependency was associated with a significantly lower ALC (median 1.15 × 10^9^/l vs. 1.26 × 10^9^/l, *p* = 0.015). Presence or absence of bone marrow fibrosis did not affect the ALC.

### Prevalence of lymphopenia, association with degree of cytopenias and impact on survival

In all, 38% of patients (*n* = 387) were lymphopenic, defined by an ALC < 1.0 × 10^9^/l. Lymphopenic patients had significantly lower counts of platelets (mean 94 × 10^9^/l vs. 129 × 10^9^/l, *p* < 0.001) and neutrophils (mean 1.33 × 10^9^/l vs. 1.81 × 10^9^/l, *p* < 0.001). The difference for the degree of anemia was less pronounced (mean hemoglobin concentration 97 g/l vs. 93 g/l, *p* = 0.014). Similar differences were noted with a cut-off of 1.2 × 10^9^/l as discriminator (see Table 1).

**Fig. 1:**
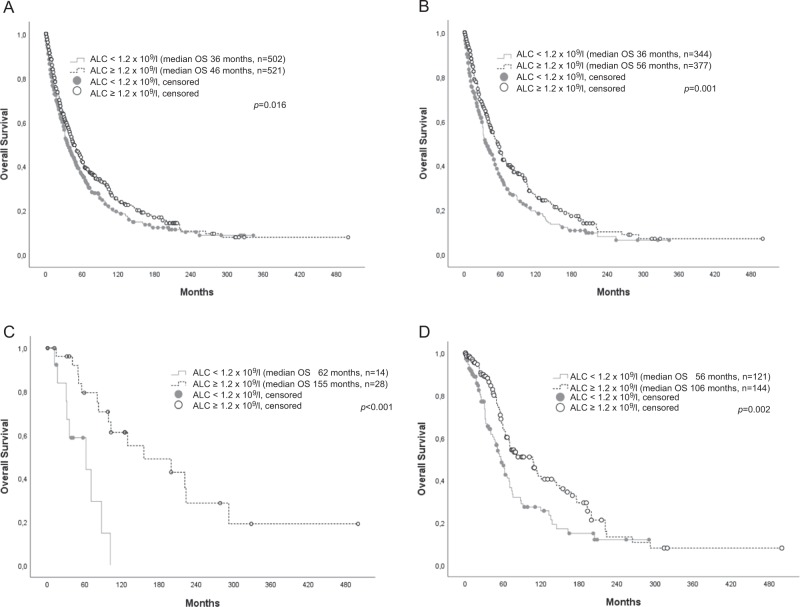
**a** Survival of an unselected MDS cohort (*n* = 1023) stratified by an ALC < or ≥1.2×10^9^/l. **b** Survival of patients with primary MDS, without induction chemotherapy or stem cell transplantation stratified by an ALC < or ≥1.2 × 10^9^/l. **c** Survival of patients with MDS-RS-SLD (*n* = 42) without induction chemotherapy or stem cell transplantation stratified by an ALC < or ≥1.2 × 10^9^/l. **d** Survival of 265 patients with primary MDS and IPSS-R low-risk MDS, without induction chemotherapy or stem cell transplantation, stratified by an ALC < or ≥1.2 × 10^9^/l

As shown in Fig. 1a, an ALC below 1.2 × 10^9^/l was associated with an inferior OS for the whole population in univariate analysis (median OS 36 vs. 46 months, *p* = 0.016; HR for death 1.21, 95% CI: 1.03–1.41, *p* = 0.017). An ALC below the normal range (<1.0 × 10^9^/l) as discriminator failed to reach statistical significance (median OS 38 vs. 43 months, *p* = 0.19; HR for death 0.9, 95% CI: 0.77–1.06, *p* = 0.194). Hence, an ALC < or ≥1.2 × 10^9^/l was used as cut-off for further survival analysis.

For patients suffering from secondary MDS or having received HSCT or induction chemotherapy OS was not influenced by lymphopenia (median OS 26 vs. 21 months, *p* = 0.89 and 46 vs. 31 months, *p* = 0.14, respectively).

Due to these findings, further survival analyses were restricted to patients suffering from primary MDS without HSCT or induction chemotherapy (*n* = 721). The influence on survival of an ALC < 1.2 × 10^9^/l in this group was highly significant (mean OS 36 vs. 56 months, *p* = 0.001; Fig. 1b). It remained an independent prognostic factor in a multivariate cox regression model together with the cytogenetic risk groups according to the IPSS-R. However, after additional inclusion of bone marrow blast count (< or ≥5%) and level of cytopenias (hemoglobin < or ≥10 g/dl, absolute neutrophil count < or ≥1.8 × 10^9^/l and platelets < or ≥100 × 10^9^/l) into the model, an ALC < 1.2 × 10^9^/l lost its additional prognostic value (*p* = 0.086, details are shown in Table 3).

**Table 3:** Factors affecting prognosis in MDS patients without hematopoietic stem cell transplantation or induction chemotherapy (*n* = 721) in univariate analysis and two multivariate models (multivariate I: absolute lymphocyte count < 1.2 × 10^9^/l together with the cytogenetic risk groups according to IPSS-R; multivariate II: absolute lymphocyte count < 1.2 × 10^9^/l together with the cytogenetic risk groups according to IPSS-R, bone marrow blast count and the degree of cytopenias)

	Univariate				Multivariate I				Multivariate II			
	*p*	Hazard ratio	95% confidence interval		*p*	Hazard ratio	95% confidence interval		*p*	Hazard ratio	95% confidence interval	
Age ≥ 65	<0.001	2.32	1.87	2.86								
Male gender	0.005	1.15	1.04	1.26								
Hb < 10 g/dl	<0.001	1.47	1.21	1.78					<0.001	1.56	1.29	1.89
Neutrophil count < 1.8 × 10^9^/l	0.001	1.39	1.15	1.67					0.959	1.0	0.82	1.23
Platelet count < 100 × 10^9^/l	<0.001	1.88	1.56	2.27					<0.001	1.56	1.28	1.89
Bone marrow blasts > 5%	<0.001	2.60	2.13	3.18					<0.001	2.25	1.82	2.79
IPSS-R cytogenetic risk category	<0.001	1.55	1.41	1.69	<0.001	1.52	1.39	1.67	<0.001	1.38	1.26	1.51
IPSS-R category	<0.001	1.68	1.55	1.82								
Transfusion dependency	0.001	1.73	1.27	2.36								
LDH > ULN (240 U/l)	<0.001	1.91	1.53	2.38								
Absolute lymphocyte count < 1.2 × 10^9^/l	0.001	1.35	1.13	1.63	0.022	1.24	1.03	1.49	0.086	0.85	0.70	1.02

With regard to the different MDS subtypes according to the WHO2016 classification, an ALC < 1.2 × 10^9^/l was associated with an inferior OS only in MDS-RS-SLD, but with a high significance despite the low patient number (*n* = 42; median OS 62 vs. 155 months, *p* = 0.001; see Fig. 1c). In all other MDS subtypes, no significant survival difference was noted (see Table 4).

**Table 4:** Impact of the absolute lymphocyte count on survival in 721 MDS patients without hematopoietic stem cell transplantation or induction chemotherapy according to MDS subtypes

	Median OS [months]			Median OS [months]		
	*n*	ALC < 1.2 × 10^9^/l	95% CI	ALC ≥ 1.2 × 10^9^/l	95% CI	*p* log rank
Whole cohort	721	36	29–43	56	48–64	0.001
*Subgroups according to WHO2016*						
MDS-SLD	57	not reached	n.a	141	26–256	0.271
MDS-MLD	227	40	26–54	44	30–58	0.433
MDS-RS-SLD	42	62	2–122	155	29–281	<0.001
MDS-RS-MLD	66	47	19–75	70	44–96	0.310
MDS del(5q)	102	62	48–76	77	47–107	0.174
MDS-EB-1	108	19	6–32	25	19–31	0.291
MDS-EB-2	109	11	6–16	22	14–30	0.268
MDS-U	10	95	n.a.	63	n.a.	0.757
*Risk categories according to IPSS-R*						
IPSS-R very low	51	101	42–160	104	71–137	0.731
IPSS-R low	265	56	46–66	106	70–142	0.002
IPSS-R intermediate	187	50	33–67	41	31–51	0.639
IPSS-R high	121	29	19–39	24	19–29	0.538
IPSS-R very high	97	8	6–10	13	9–17	0.127

After stratification according to IPSS-R, the additional prognostic value of an ALC < 1.2 × 10^9^/l was restricted to the IPSS-R low-risk group (median OS 56 vs. 106 months, *p* = 0.002; see Fig. 1d). It kept its independent prognostic value for this patient subset after inclusion into a multivariable Cox regression model together with age < or ≥65 years and LDH < or ≥normal value (see Table 5).

**Table 5:** Additional prognostic value of different factors for patients within the IPSS-R low-risk group without hematopoietic stem cell transplantation or induction chemotherapy (*n* = 265)

	Unvariate				Multivariate			
	*p* Value	Hazard ratio	95% CI		*p* Value	Hazard ratio	95% CI	
Age > 65	<0.001	3.31	2.25	4.88	<0.001	3.21	2.15	4.80
LDH > ULN (240 U/l)	<0.001	2.31	1.51	3.53	<0.001	2.58	1.67	3.98
Absolute lymphocyte count <1.2 × 10^9^/l	0.002	1.68	1.21	2.34	0.033	1.46	1.03	2.08
Male gender	0.054	1.39	0.99	1.93				
Transfusion dependency	0.075	1.61	0.95	2.71				
Bone marrow fibrosis	0.691	0.87	0.45	1.69				
Hb < 10 g/dl	0.287	1.20	0.86	1.66				
Neutrophil count < 1.8 × 10^9^/l	0.148	0.79	0.57	1.09				
Platelet count < 100 × 10^9^/l	0.174	0.73	0.47	1.15				
Bone marrow blasts > 5%	0.916	1.04	0.49	2.23				
IPSS-R cytogenetic risk category	0.213	1.44	0.81	2.57				

An ALC < 1.2 × 10^9^/l was not associated with a shorter leukemia-free survival (mean 22.4 vs. 32.8 months, *p* = 0.859).

## Discussion

Analyzing a large cohort of 1023 uniformly characterized patients, we found evidence for a significant impairment of lymphopoiesis in MDS. Totally, 38% of patients were lymphopenic (ALC < 1.0 × 10^9^/l) and the median lymphocyte count of 1.22 × 10^9^/l observed in our cohort was conssiderably lower than the median ALC of about 2.0 × 10^9^/l, published for a large cohort of healthy, nonhispanic caucasian US Americans in the corresponding age groups^10^. In addition, an ALC < 1.2 × 10^9^/l was associated with a worse OS in univariate analysis.

These results are consistent with previously published data on lymphopenia in MDS and its impact on survival. A prognostic role for an ALC < 1.2 × 10^9^/l in MDS with del(5q), including patients with an increased blast count and additional cytogenetic abnormalities, was first reported by Holtan et al.^6^. Subsequently, a lymphopenia defined by this cut-off was considered to be an independent prognostic factor in addition to IPSS and WPSS in MDS without del(5q) as well^7^. Within an unselected cohort of primary MDS, an ALC < 1.2 × 10^9^/l was associated with an inferior OS in univariate analysis but failed to reach statistical significance, when including the more refined cytogenetic risk categories according to IPSS-R into a multivariate Cox regression model^8^.

In addition to the confirmation of these previous observations in an independent cohort, our findings add some new aspects to the role of lymphopenia in MDS. First, the additional prognostic value of a low-lymphocyte count seems to be restricted to low-risk patients, if the IPSS-R is used for risk stratification. However, for this subgroup it can be considered as a relative strong additional prognostic marker, since it retained its independent prognostic value in a multivariate Cox regression model together with age and an elevated LDH, two additional risk factors well-known to affect prognosis in MDS^2,11^.

The lack of an additional prognostic value for the IPSS-R very low-risk category in our cohort may be due to the very low patient number in this cohort (*n* = 51). Biology and clinical course of high-risk and very high-risk MDS are primarily characterized by genomic instability and AML evolution. In this setting, an additional impairment of the adaptive immune system does not seem to have additional prognostic value.

Alterations of both the innate and the adaptive immune system have long been recognized as a feature in MDS^12,13^. The T-cell compartment has been most extensively studied, with a focus on its involvement in the pathophysiology of MDS. To date, it is unknown, whether lymphopenic MDS patients differ from MDS patients with a normal lymphocyte count with regard to the distribution or functional capacity of different lymphocyte subsets. Whether lymphopenia in MDS is a direct consequence of underlying hematopoietic stem cell defect(s) or arises from immune-modulating stimuli related to the disease or to other host conditions remains to be elucidated as well.

Here, some of our observations point towards a direct relationship between MDS pathophysiology and low lymphocyte counts. In our cohort, neither age nor comorbidities were associated with a lower ALC and significant differences with regard to the ALC were noted between different MDS subtypes. Notably, ALCs were lower in subtypes with a worse prognosis, like higher-risk MDS according to the IPSS-R, cases of therapy related MDS and in patients being transfusion dependent at diagnosis. In addition, lymphopenia was associated with lower peripheral blood counts as markers of disease severity, a finding that has been described previously^8^.

Some of the key features of T-cell alterations seen in aging individuals^14^ have also been described as features of T-cell lymphopoiesis in MDS, as for example a skewed T-cell receptor repertoire^15^ and a contracted pool of CD8+ T cells^16^. Hence, the overlapping features of the physiology of aging hematopoiesis and the pathophysiology of MDS, which have come into focus recently^17^, may provide some explanations for the lymphopenia occurring in MDS-patients.

Many changes of the adoptive immune system in the elderly, often referred to as immunosenescence, can be directly traced back to changes in the stem cell compartment, which typically shows a myeloid-skewing with a diminished capacity for lymphoid differentiation^18^. Subclinical inflammatory changes in the bone marrow microenvironment are a further hallmark of the aging hematopoietic stem cell niche^19^. Recently, microinflammation in the bone marrow mediated by the NLRP3 inflammasome has been identified as one driver of the MDS phenotype^20^ and activation of the NLRP3-inflammasome within the bone marrow is known to inhibit B-cell lymphopoiesis at least in vitro^21^. Additional alterations in the bone marrow niche linking impairment of lymphopoiesis and inflammatory changes likely exist and may not only cause ineffective hematopoiesis, but also compromise homeostasis of both B and T cells.

Furthermore, lymphocytes can be part of the MDS-clone. For MDS with del(20q)^22^, Monosomy 7 (ref. ^23^), trisomy 8 (ref. ^24^) and del(5q)^23^, the respective cytogenetic anomalies have been described in B- and/or T-cell lymphocytes, leading to the hypothesis that the MDS clones arise in a common myeloid and lymphoid progenitor at least in some cases. This was demonstrated in a study applying NGS to bone marrow cell populations sorted according to their differentiation stage in MDS-RS-SLD^24^. The initiating SF3B1 mutation was found in a lympho-myeloid progenitor and is consistently detectable at the pro-B-cell progenitor stage. Since the mutation was rarely observed in mature B cells, it can be concluded that SF3B1-mutations are likely to impair B-cell development.

Concerning T-cell involvement in myeloid neoplasms, mutations in the epigenetic regulator DNMT3A are detectable in AML patients in both leukemic blasts and in T cells at diagnosis^25^, and may persist in first complete remission in both compartments^26^. Since DNMT3A has recently been shown to be involved in T-cell development^27^, DNMT3A mutations may affect T-cell biology and due to the many pathways shared between MDS and AML, MDS could be derived from lympho-myeloid clonal hematopoiesis at least in some subtypes. However, the clonal involvement of T cells in MDS remains to be studied systematically.

Both Lenalidomide^28,29^, and hypomethylating agents^30^ alter lymphocyte biology during treatment. Hence, these treatments could possibly be able to reverse the negative prognostic impact of a lymphopenia at diagnosis. Only a limited number of patients in our cohort were treated with these compounds, so we are not able to address the question whether a low ALC remains a significant prognostic factor in patients treated with Lenalidomide and hypomethylating agents. Further studies addressing this question are needed. Since the ALC is readily available in clinical routine this question can be addressed in clinical trials or register studies. Furthermore, the additional prognostic value of the ALC should be investigated after refinement of the IPSS-R by the inclusion of adverse molecular features revealed by NGS^31^.

Taken together, our data support the hypothesis of subtle but clinically relevant changes of the adaptive immune system in MDS. Further studies are necessary to define the ALC cut-off most suitable for prognostication and to identify the mechanisms underlying the impairment of lymphoid homeostasis in MDS.
